# Erratum for Zhang et al., “Source Tracking and Global Distribution of the Tigecycline-Nonsusceptible Tet(X)”

**DOI:** 10.1128/spectrum.01131-22

**Published:** 2022-04-27

**Authors:** Rong-min Zhang, Jian Sun, Ruan-yang Sun, Min-ge Wang, Chao-yue Cui, Liang-xing Fang, Mei-na Liao, Xiao-qing Lu, Yong-xin Liu, Xiao-Ping Liao, Ya-Hong Liu

**Affiliations:** a National Risk Assessment Laboratory for Antimicrobial Resistance of Animal Original Bacteria, South China Agricultural University, Guangzhou, China; b Laboratory of Veterinary Pharmacology, College of Veterinary Medicine, South China Agricultural University, Guangzhou, China; c Guangdong Laboratory for Lingnan Modern Agriculture, Guangzhou, People’s Republic of China; d State Key Laboratory of Plant Genomics, Institute of Genetics and Developmental Biology, Innovation Academy for Seed Design, Chinese Academy of Sciences, Beijing, China

## ERRATUM

Volume 9, no. 3, 2021, https://doi.org/10.1128/Spectrum.01164-21. Page 1: The title should appear as given in this Erratum.

Page 12, Acknowledgments: The first paragraph should read as follows. “This work was supported by the Natural Science Foundation of Guangdong Province, China (2022A1515011532), Foundation for Innovative Research Groups of the National Natural Science Foundation of China (32121004), Local Innovative and Research Teams Project of Guangdong Pearl River Talents Program (2019BT02N054), and Innovation Team Project of Guangdong University (2019KCXTD001).”

