# Supplementary material for: Source Tracking and Global Distribution of the Tigecycline Non-Susceptible tet(X)
Source: Microbiol Spectr. 2021 Dec 22;9(3):e01164-21. doi: 10.1128/Spectrum.01164-21 (PMC8693923; doi:10.1128/Spectrum.01164-21)
Supplement: SUPPLEMENTAL FILE 2 — Supplemental material. Download SPECTRUM01164-21_Supp_1_seq9.pdf, PDF file, 2.1 MB [file spectrum01164-21_supp_1_seq9.pdf]

Supplementary file legends

Text S1. A total of 202,265 MAGs that was constructed from the 12,829 non-duplicate metagenomic samples.

Text S2. Description of the comparison of *tet*(X) genomic-environment in Figure 4 and S5.

Figure S1. Phylogenetic analysis of the Tet(X) at amino acid level.

Figure S2. Comparative analysis of the Tet(X2)-like orthologs at the amino acid level.

Figure S3. Significance analysis of *tet*(X2) distributions in Europe, Asia and America.

Figure S4. Distribution of *tet*(X2)-like orthologs among different bacterial genus, countries and age groups from microbial genomic bins of human-gut origin.

Figure S5. Comparative analysis of the *tet*(X2) genomic context among *E. coli*, *R. anatipestifer*, *Phocaeicola vulgatus* and *Odoribacter laneus*. Arrows indicate the directions of transcription of the genes, and different genes are shown in different colors. Regions of ≥ 99.0% nucleotide sequence identity are shaded light grey. The *Δ* symbol indicates a truncated gene. IS, insertion sequence. See Table S11 for genomic Type XVIII – XXII definitions.

Table S1. A total of 12,829 non-duplicate metagenomic samples that derived from previous studies (see PMID of Publication).

Table S2. A total of 322 *tet*(X) positive MAGs in the 202,265 MAGs.

Table S3. The *tet*(X2)-like and non *tet*(X2) orthologs designed in current study.

Table S4. Minimum inhibitory concentration of the *tet*(X)s from metagenomic analysis. TET: tetracycline; DOX: doxycycline; MIN; minocycline; TIG: tigecycline; ERA: eravacycline; OMA: omadacycline.

Table S5. Positive rates of *tet*(X) gene in 31 countries.

Table S6. Positive rates of *tet*(X) carrying MAGs annotated at family level.

Table S7. Positive rates of *tet*(X) carrying MAGs annotated at species level.

Table S8. Detail information of the 322 *tet*(X) carrying MAGs.

Table S9. Detail information of the 896 *tet*(X) carrying bacterial isolates.

Table S10. Clusters of the 1218 *tet*(X) positive contigs from the MAGs and bacterial isolates.

Table S11. Detail information of the *tet*(X) genomic context types I to XXII.

Text S2

Compared with genomic type I - III (Figure 4b), an original genomic structure before the insertion of *tet*(X)s in type II was found from a *Bacteroides* sp. isolate recovered from human gut (Figure 4b). In the downstream of type I genomic context, an IS*Bf11* element located at the downstream of *tet*(X45.2) and IS*Bf11* contained classical left inverted repeats (IRL) according to the ISfinder database [1] but the right inverted repeats (IRR) was missing. A short flanking direct target DNA repeats (DRs: aagtacc) located immediately upstream of the IRL and 37-bp downstream of IS*Bf11*. These elements formed a trail of integrated region DR-IRL-IS*Bf11*-DR. Coincidently, the1880 bp upstream fragment (bla_{OXA347}-*erm*(F)) and 2805 bp downstream fragment (*rfa*H-hp-hp-hp) of this integrated region in type I genomic context s shared more than 99% similarity to a serial nucleotide sequences in type II. This suggests that the IS*Bf11* inserted into the downstream of *erm*(F) and formed a genomic array *erm*(F)-IS*Bf11*-*rfa*H-*hp-hp-hp*, which was also present at the downstream of *tet*(X2.4) in type III. The IS*4351* was another IS element closely flanking *tet*(X2.4) in type III. The classic IRL and IRR bracketing IS*4351* (IRL-IS*Bf11*-IRR) were also identified according to the ISfinder and the sequences immediately upstream and downstream of this region were identical to a serial nucleotide sequences that contained *tet*(X47) in genomic type IV (Figure 4b). This indicates that the IS*4351* could insert upstream of *tet*(X)s. Type VI, VII and VIII genomic structures including *tet*(X46.2), *tet*(X2.4) and *tet*(X46), respectively, were closely resembling among them with high similarity (>97%) and coverage (>90%) (Figure 4b). This suggested that the *tet*(X2)-like orthologs, especially *tet*(X2.4), were ready to mutate and form tigecycline resistant non-*tet*(X2) orthologs. Thus, a possible formation of these tigecycline resistant non-*tet*(X2) orthologs suggested that the IS*4351* and IS*Bf11* bracketing the *tet*(X2)-*erm*(F)

formed a transient transposon, and this structure was able to integrate into the *erm*(F) associated region (Figure 4b). It was noteworthy that I - VII Type genomic contexts were from MGBs annotated as anaerobe, excluding *tet*(X46.2) in type VIII that was carried by a MGB annotated as *Enterococcus faecalis* from Italy.

All of genomic context types IX, X and XI were found in facultative anaerobic *Riemerella anatipestifer* isolates. Genomic type IX covered all of the ORFs presented in type VIII, although their relative locations were disarranged by other ORF (Figure 4b). Two 40 kb nucleotide sequences located upstream of the genomic context types IX and X shared more than 99% identity. In their downstream regions, two copies of reverse repeat sequences *bla*$_{OXA209}$-*rmdc* and *rmdc*-*bla*$_{OXA209}$ were observed and they bracketed a multiple-drug resistant genomic region *flo*R-*erm*(F)-*hp*-*hp*-△*aad*K that was absent in genomic type X (Figure 4b).

The *tet*(X3) in genomic context type XIII and *tet*(X4) in XV were from *Acinetobacter* sp. and *Aeromonas caviae*, respectively [2], and the genomic array *rdm*C-*tet*(X4)-△IS*CR2* in genomic context XVI also existed in *E. coli*, *Acinetobacter* sp. and *Salmonella* from a variety of hosts (Figure 3b). The nucleotide sequences flanking these two most prevalent *tet*(X)s shared a high similarity with their corresponding region from aerobes and facultative anaerobes, including *Flavobacterium* in type XII, *Riemerella anatipestifer in* type XIV, *Myroides phaeus* in type XVI and *Chryseobacterium* type XVIII (Figure 4b), although the *tet*(X) in these isolates shared only 77.23% - 79.06% and 84.47% - 90.79% similarity with the *tet*(X3) and *tet*(X4) respectively (Figure 4b). IS*CR2* existed in all of these genomic contexts and closely flanked *tet*(X). In genomic type XVI, IS*CR2* was located immediately downstream of the *tet*(X), and only one or two ORFs embedded between IS*CR2* and *tet*(X) in other genomic context. These indicated that the

non-*tet*(X2) orthologs could spread between *Flavobacteriaceae* and the *tet*(X3/4) carriers and IS*CR2* played an important role in the transmission.

We found the *tet*(X2) orthologs distributed in only two *E. coli* isolates in our study, and their genomic contexts were presented in genomic type XXI and XXII (Figure S5). An array *tet*(X2)-*hp*-△IS*Bbi1* included in a 2,014-bp nucleotide sequence from *E. coli* was identical to the corresponding region from *Odoribacter laneus*, but the △IS*Bbi1* located downstream of this array was only remaining 76 bp in type XXI. The genomic array *aad*S-*ere*(D)-*tet*(X2) not only presented in *Odoribacter laneus*, but also in a *Phocaeicola vulgatus* (type XIX) isolate. The downstream *ere*(D)-*tet*(X2) also presented in our earliest emerged tet(X) positive *R. anatipestifer* isolates collected in 1966 (type XVIII).

[1] Siguier P, Perochon J, Lestrade L, et al. Isfinder: The reference centre for bacterial insertion sequences. Nucleic Acids Res 2006;34:D32-36.

[2] Chen C, Cui C-Y, Yu J-J, et al. Genetic diversity and characteristics of high-level tigecycline resistance tet(X) in acinetobacter species. Genome Med 2020;12:111.

Figure S1

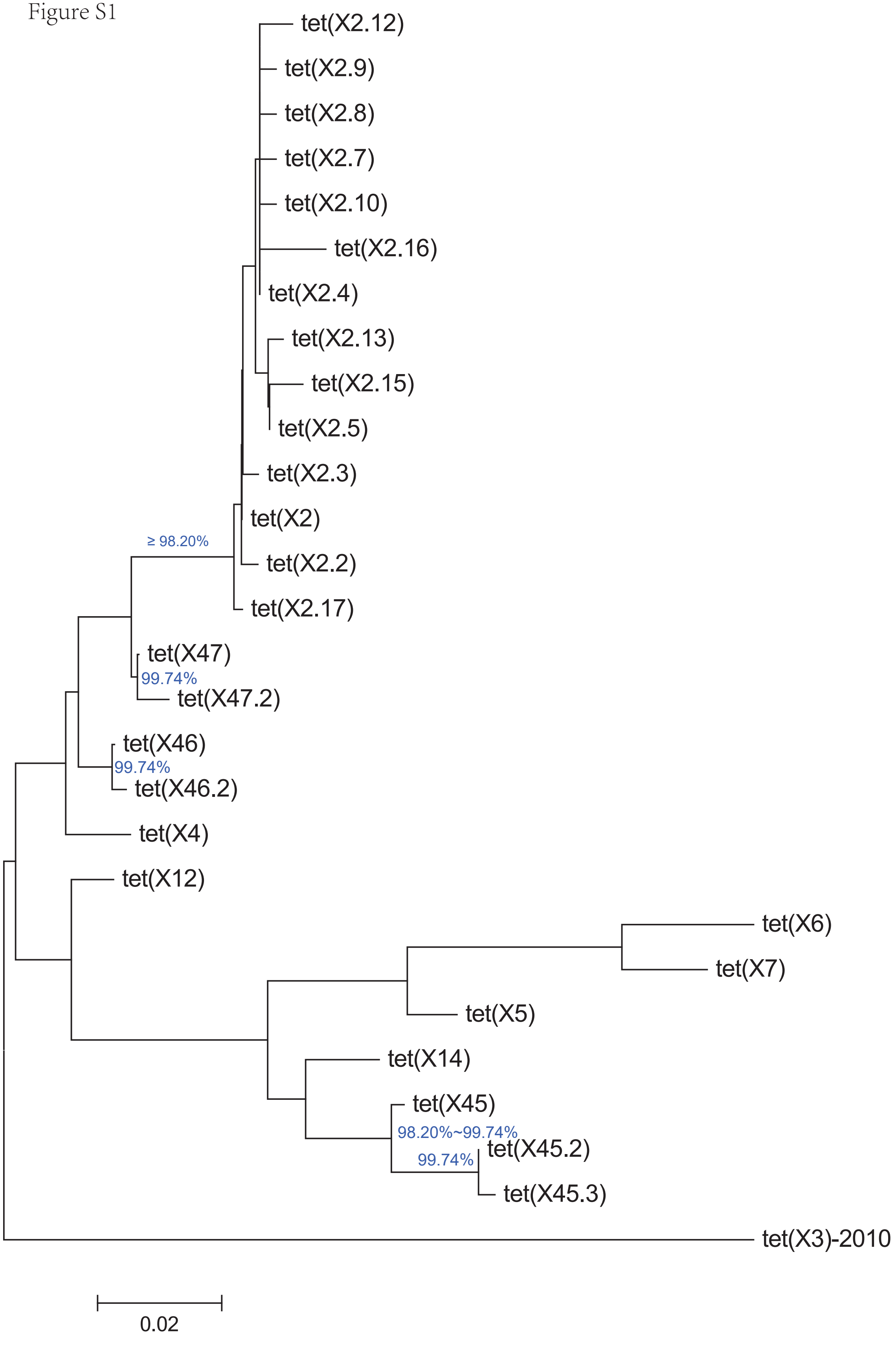
tet(X2.12)
tet(X2.9)
tet(X2.8)
tet(X2.7)
tet(X2.10)
tet(X2.16)
tet(X2.4)
tet(X2.13)
tet(X2.15)
tet(X2.5)
tet(X2.3)
tet(X2)
≥ 98.20%
tet(X2.2)
tet(X2.17)
tet(X47)
99.74%
tet(X47.2)
tet(X46)
99.74%
tet(X46.2)
tet(X4)
tet(X12)
tet(X6)
tet(X7)
tet(X5)
tet(X14)
tet(X45)
98.20%~99.74%
tet(X45.2)
99.74%
tet(X45.3)
tet(X3)-2010
0.02

Figure S2

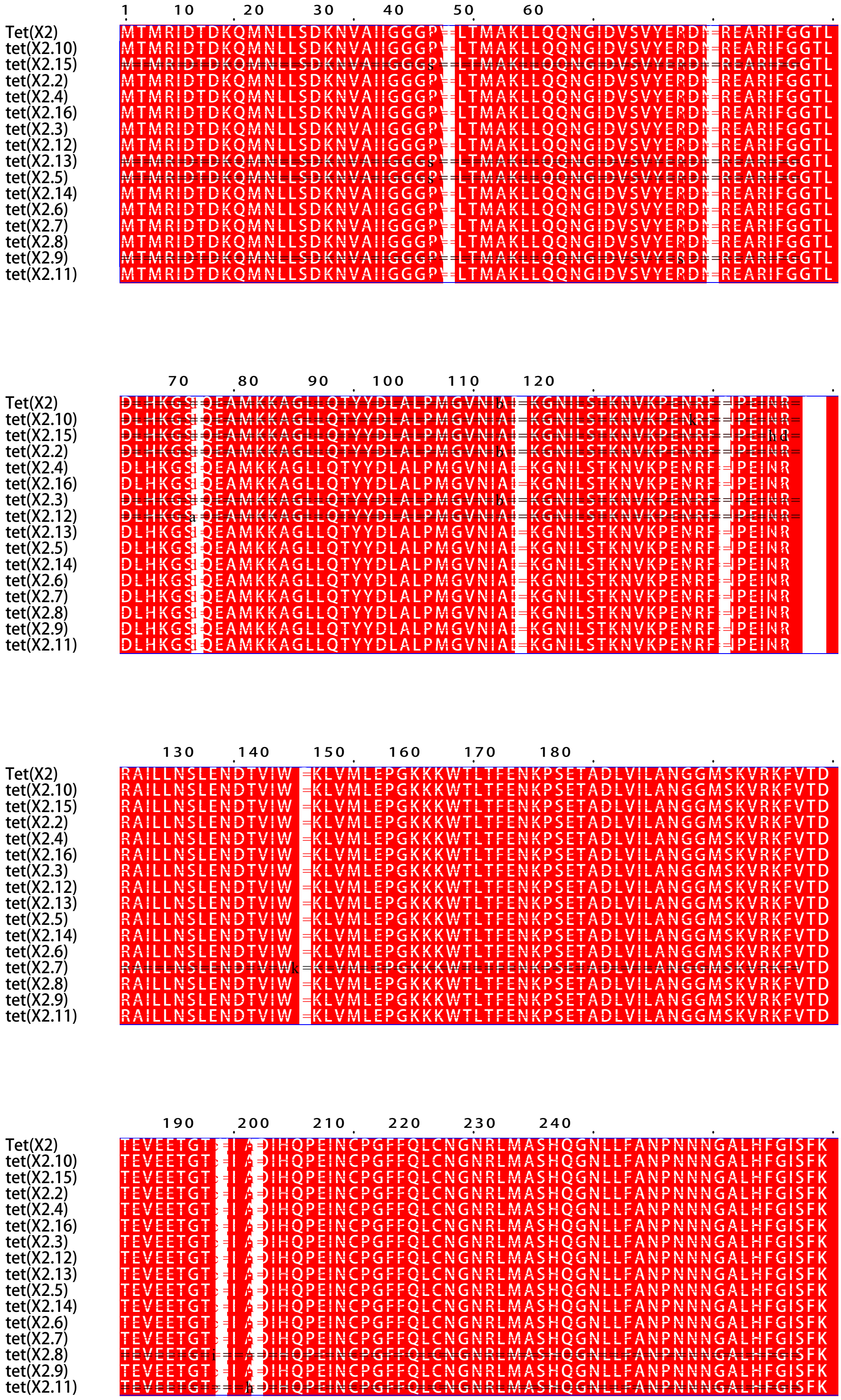

```
                      250       260       270       280       290       300
Tet(X2)         TPDEWKNQTQVDFQNRNSVVDFLLKEFSDWDERYKELIHTTLSFVGLATRIFPLEKPWKS
tet(X2.10)      TPDEWKNQTQVDFQNRNSVVDFLLKEFSDWDERYKELIHTTLSFVGLATRIFPLEKPWKS
tet(X2.15)      TPDEWKNQTQVDFQNRNSVVDFLLKEFSDWDERYKELIHTTLSFVGLATRIFPLEKPWKS
tet(X2.2)       TPDEWKNQTQVDFQNRNSVVDFLLKEFSDWDERYKELIHTTLSFVGLATRIFPLEKPWKS
tet(X2.4)       TPDEWKNQTQVDFQNRNSVVDFLLKEFSDWDERYKELIHTTLSFVGLATRIFPLEKPWKS
tet(X2.16)      TPDEWKNQTQVDFQNRHTVVDFLLKEFSDWDERYKELIHTTLSFVGLATRIFPLEKHTKS
tet(X2.3)       TPDEWKNQTQVDFQNRNSVVDFLLKEFSDWDERYKELIHTTLSFVGLATRIFPLEKPWKS
tet(X2.12)      TPDEWKNQTQVDFQNRNSVVDFLLKEFSDWDERYKELIHTMLSFVGLATRIFPLEKPWKS
tet(X2.13)      TPDEWKNQTQVDFQNRNSVVDFLLKEFSDWDERYKELIHTTLSFVGLATRIFPLEKPWKS
tet(X2.5)       TPDEWKNQTQVDFQNRNSVVDFLLKEFSDWDERYKELIHTTLSFVGLATRIFPLEKPWKS
tet(X2.14)      TPDEWKNQTQVDFQNRNSVVDFLLKEFSDWDERYKELIHTTLSFVGLATRIFPLEKPWKS
tet(X2.6)       TPDEWKNQTQVDFQNRNSVVDFLLKEFSDWDEHYKELIHTTLSFVGLATRIFPLEKPWKS
tet(X2.7)       TPDEWKNQTQVDFQNRNSVVDFLLKEFSDWDERYKELIHTTLSFVGLATRIFPLEKPWKS
tet(X2.8)       TPDEWKNQTQVDFQNRNSVVDFLLKEFSDWDERYKELIHTTLSFVGLATRIFPLEKPWKS
tet(X2.9)       TPDEWKNQTQVDFQNRNSVVDFLLKEFSDWDERYKELIHTTLSFVGLATRIFPLEKPWKS
tet(X2.11)      TPDEWKNQTQVDFQNRNSVVDFLLKEFSDWDERYKELIHTTLSFVGLATRIFPLEKPWKS
```

```
                      310       320       330       340       350       360
Tet(X2)         KRPLPITMIGDAAHLMPPFAGQGVNSGLVDALILSDNLADGKFNSIEEAVKNYEQQMFIY
tet(X2.10)      KRPLPITMIGDAAHLMPPFAGQGVNSGLVDALILSDNLADGKFNSIEEAVKNYEQQMFIY
tet(X2.15)      KRPLPITMIGDAAHLMPPFAGQGVNSGLVDALILSDNLADGKFNSIEEAVKNYEQQMFIY
tet(X2.2)       KRPLPITMIGDAAHLMPPFAGQGVNSGLVDALILSDNLADGKFNSIEEAVKNYEQQMFIY
tet(X2.4)       KRPLPITMIGDAAHLMPPFAGQGVNSGLVDALILSDNLADGKFNSIEEAVKNYEQQMFIY
tet(X2.16)      KRPLPITMIGDAAHLMPPFAGQGVNSGLVDALILSDNLADGKFNSIEEAVKNYEQQMFIY
tet(X2.3)       KRPLPITMIGDAAHLMPPFAGQGVNSGLVDALILSDNLADGKFNSIEEAVKNYEHQMFIY
tet(X2.12)      KRPLPITMIGDAAHLMPPFAGQGVNSGLVDALILSDNLADGKFNSIEEAVKNYEQQMFIY
tet(X2.13)      KRPLPITMIGDAAHLMPPFAGQGVNSGLVDALILSDNLADGKFNSIEEAVKNYEHQMFIY
tet(X2.5)       KRPLPITMIGDAAHLMPPFAGQGVNSGLVDALILSDNLADGKFNSIEEAVKNYEQQMFIY
tet(X2.14)      KRPLPITMIGDAAHLMPPFAGQGVNSGLVDALILSDNLADGKFNSIEETVKNYEQQMFIY
tet(X2.6)       KRPLPITMIGDAAHLMPPFAGQGVNSGLVDALILSDNLADGKFNSIEEAVKNYEQQMFIY
tet(X2.7)       KRPLPITMIGDAAHLMPPFAGQGVNSGLVDALILSDNLADGKFNSIEEAVKNYEQQMFIY
tet(X2.8)       KRPLPITMIGDAAHLMPPFAGQGVNSGLVDALILSDNLADGKFNSIEEAVKNYEQQMFIY
tet(X2.9)       KRPLPITMIGDAAHLMPPFAGQGVNSGLVDALILSDNLADGKFNSIEEAVKNYEQQMFIY
tet(X2.11)      KRPLPITMIGDAAHLMPPFAGQGVNSGLVDALILSDNLADGKFNSIEEAVKNYEQQMFIY
```

```
                      370       380
Tet(X2)         GKEAQEESTQNEIEMFKPDFTFQQLLNV
tet(X2.10)      GKEAQEESTQNEIEMFKPDFTFQQLLNV
tet(X2.15)      GKEAQEESTQNEIEMFKPDFTFQQLLNV
tet(X2.2)       GKEAQEESTQNEVEMFKPDFTFQQLLNV
tet(X2.4)       GKEAQEESTQNEIEMFKPDFTFQQLLNV
tet(X2.16)      GKEAQEESTQNEIEMFKPDFTFQQLLNV
tet(X2.3)       GKEAQEESTQNEIEMFKPDFTFQQLLNV
tet(X2.12)      GKEAQEESTQNEIEMFKPDFTFQQLLNV
tet(X2.13)      GKEAQEESTQNEIEMFKPDFTFQQLLNV
tet(X2.5)       GKEAQEESTQNEIEMFKPDFTFQQLLNV
tet(X2.14)      GKEAQEESTQNEIEMFKPDFTFQQLLNV
tet(X2.6)       GKEAQEESTQNEIEMFKPDFTFQQLLNV
tet(X2.7)       GKEAQEESTQNEIEMFKPDFTFQQLLNV
tet(X2.8)       GKEAQEESTQNEIEMFKPDFTFQQLLNV
tet(X2.9)       GKEAQEESTQNEIEMFKPDFTFQQLLNV
tet(X2.11)      GKEAQEESTQNEIEMFKPDFTFQQLLNV
```

Figure S3

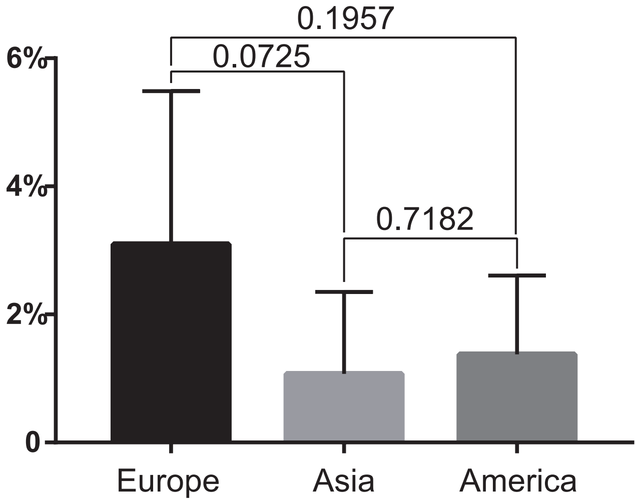

Figure S4

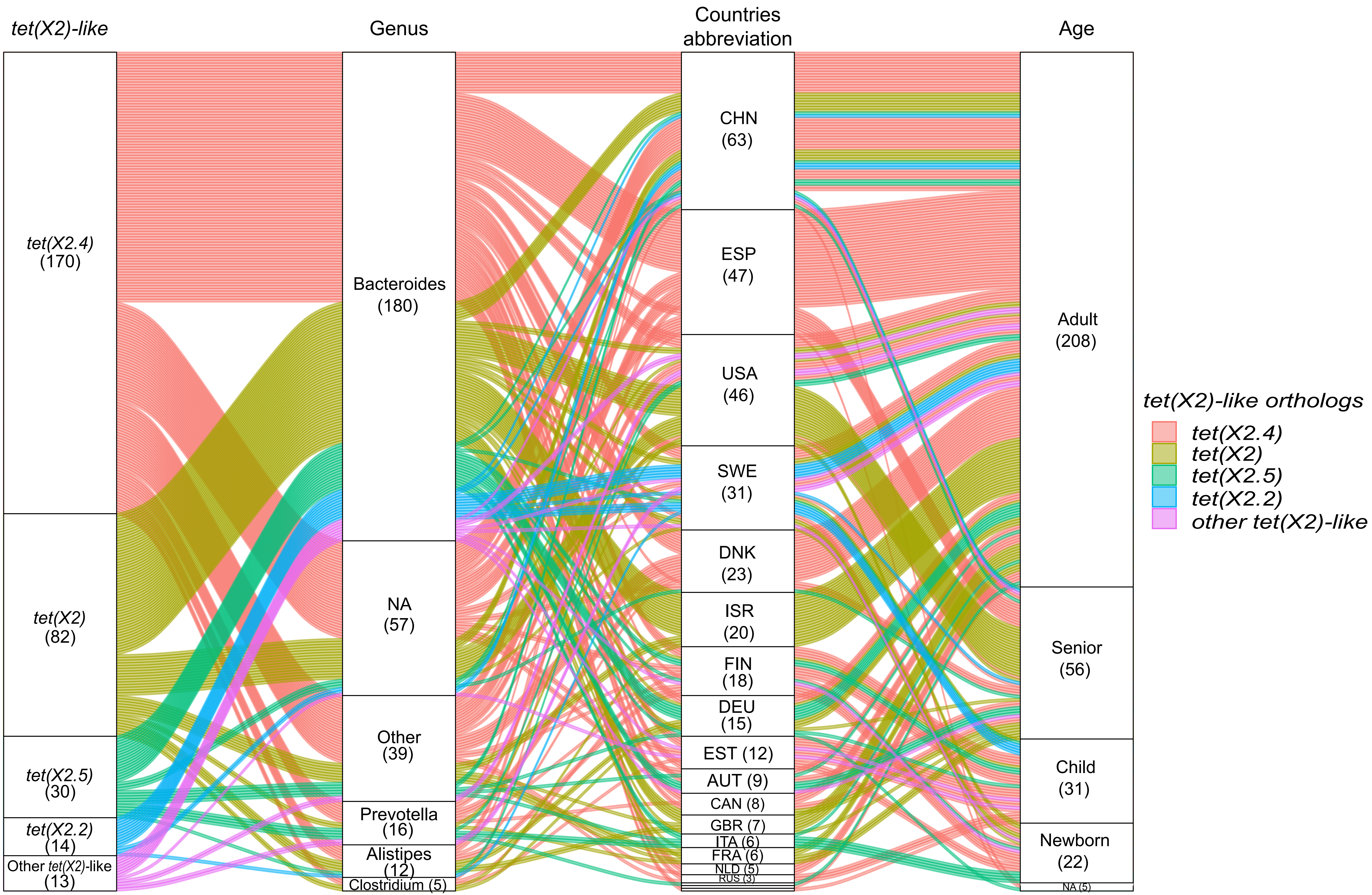

tet(X2)-like
Genus
Countries abbreviation
Age
tet(X2.4) (170)
tet(X2) (82)
tet(X2.5) (30)
tet(X2.2) (14)
Other tet(X2)-like (13)
Bacteroides (180)
NA (57)
Other (39)
Prevotella (16)
Alistipes (12)
Clostridium (5)
CHN (63)
ESP (47)
USA (46)
SWE (31)
DNK (23)
ISR (20)
FIN (18)
DEU (15)
EST (12)
AUT (9)
CAN (8)
GBR (7)
ITA (6)
FRA (6)
NLD (5)
RUS (3)
Adult (208)
Senior (56)
Child (31)
Newborn (22)
NA (5)
tet(X2)-like orthologs
tet(X2.4)
tet(X2)
tet(X2.5)
tet(X2.2)
other tet(X2)-like

Figure S5

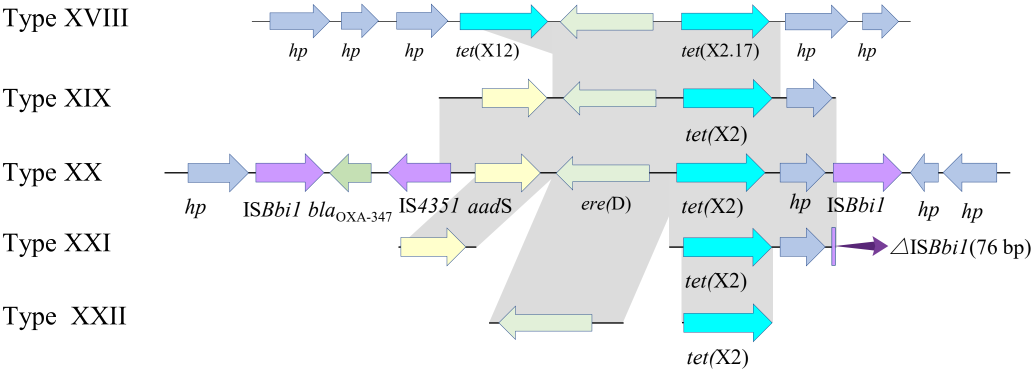
Type XVIII
hp
hp
hp
tet(X12)
tet(X2.17)
hp
hp
Type XIX
tet(X2)
Type XX
hp
ISBbi1
bla OXA-347
IS4351
aadS
ere(D)
tet(X2)
hp
ISBbi1
hp
hp
Type XXI
tet(X2)
△ISBbi1(76 bp)
Type XXII
tet(X2)
